# Lessons Learned from Multiobjective Automatic Optimizations of Classical Three-Site Rigid Water Models Using Microscopic and Macroscopic Target Experimental Observables

**DOI:** 10.1021/acs.jced.3c00538

**Published:** 2023-12-05

**Authors:** Mattia Perrone, Riccardo Capelli, Charly Empereur-mot, Ali Hassanali, Giovanni M. Pavan

**Affiliations:** † Department of Applied Science and Technology, Politecnico di Torino, Corso Duca degli Abruzzi 24, Torino I-10129, Italy; ‡ Department of Biosciences, Università degli Studi di Milano, Via Celoria 26, Milano I-20133, Italy; § Department of Innovative Technologies, University of Applied Sciences and Arts of Southern Switzerland, Polo Universitario Lugano, Campus Est, Via la Santa 1, Lugano-Viganello CH-6962, Switzerland; ∥ The Abdus Salam International Center for Theoretical Physics, Strada Costiera 11, Trieste 34151, Italy

## Abstract

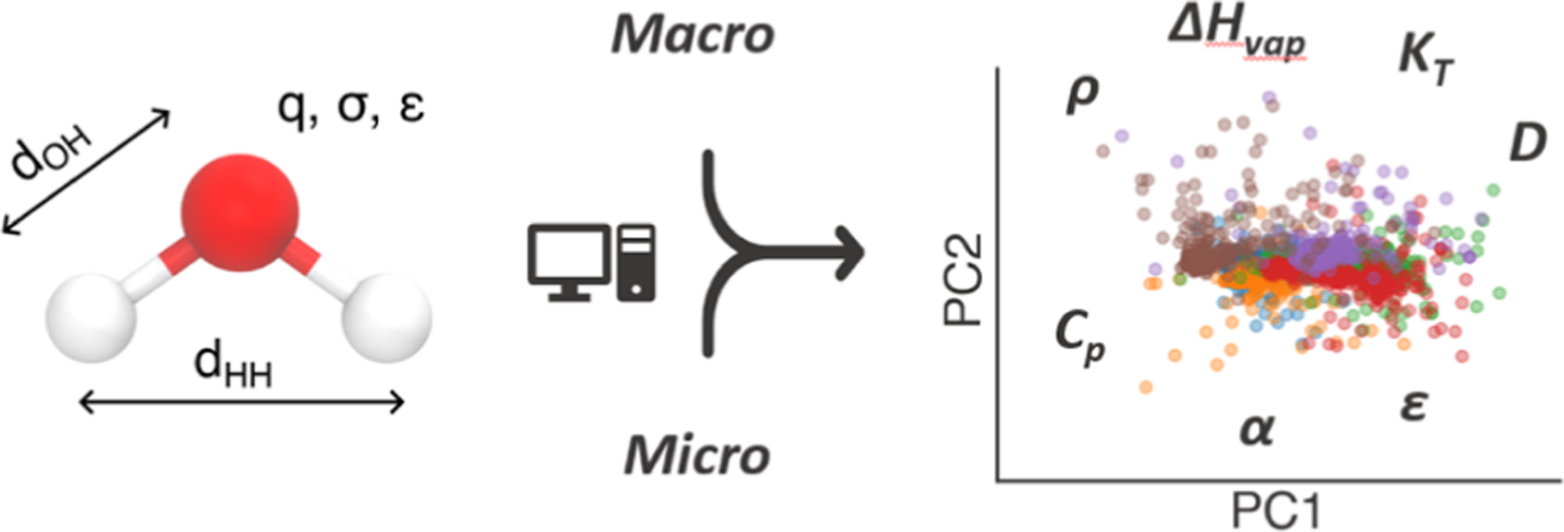

The development of accurate water models is of primary importance for molecular simulations. Despite their intrinsic approximations, three-site rigid water models are still ubiquitously used to simulate a variety of molecular systems. Automatic optimization approaches have been recently used to iteratively refine three-site water models to fit macroscopic (average) thermodynamic properties, providing state-of-the-art three-site models that still present some deviations from the liquid water properties. Here, we show the results obtained by automatically optimizing three-site rigid water models to fit a combination of microscopic and macroscopic experimental observables. We use *Swarm-CG*, a multiobjective particle-swarm-optimization algorithm, for training the models to reproduce the experimental radial distribution functions of liquid water at various temperatures (rich in microscopic-level information on, e.g., the local orientation and interactions of the water molecules). We systematically analyze the agreement of these models with experimental observables and the effect of adding macroscopic information to the training set. Our results demonstrate how adding microscopic-rich information in the training of water models allows one to achieve state-of-the-art accuracy in an efficient way. Limitations in the approach and in the approximated description of water in these three-site models are also discussed, providing a demonstrative case useful for the optimization of approximated molecular models, in general.

## Introduction

The development and optimization of classical molecular models is typically challenging and time-consuming.^1,2^ Despite notable progresses in developing efficient methods and optimization approaches,^3−9^ accurately predicting experimental observables and ensuring transferability across varying thermodynamics conditions remains in most cases a significant challenge.^10−12^ A considerable example is the case of water, for which current state-of-art models struggle in matching all the relevant cases of interest at the same time,^13^ e.g., bulk properties,^14^ free energy of hydration of compounds,^15^ stabilization of lipid membranes,^16^ interaction with proteins,^17^*etc.*

Although intrinsically approximated, classical three-site rigid water models are widely used in molecular dynamics (MD) simulations.^14^ One key requirement is that such simplified models can capture fairly well the properties of water, even relying on a reduced number of parameters. In such a representation, the interaction potential is centered on three sites, each of which corresponds to one of the atoms in the water molecule (O, H, and H). Early versions of these models, including, e.g., TIP3P^18^ and SPC,^19^ were originally parameterized to accurately reproduce basic thermodynamic properties, e.g., density and enthalpy of vaporization under standard conditions. Despite their age, these models continue to be extensively utilized in classical MD simulations, and most general-purpose force fields are parametrized on them.^20−22^ With the increase in computing power, it has become possible to perform high-throughput parametrization, often in an automatic fashion,^23,24^ by considering a large set of experimental observables under different conditions as the reference data to fit.

Over the past decade, two notable general-purpose three-site water models that have been obtained through iterative optimization, TIP3P-FB,^25^ and OPC3,^26^ have led to a substantial improvement of the state of the art. Such models were refined to accurately reproduce a set of thermodynamic properties, including density, heat of vaporization, coefficient of thermal expansion, isothermal compressibility, isobaric heat capacity, and static dielectric constant. In particular, TIP3P-FB was optimized to accurately reproduce these observables over a wide range of thermodynamic conditions, spanning a total of 40 training points at different temperatures and pressures. Such a parallel/multiobjective parameterization has a positive effect on the transferability of the optimized model,^27^ e.g., across different conditions. In contrast, OPC3 was optimized to match such observables under standard conditions (298 K and 1 bar) while simultaneously imposing a constraint on the geometry of the water molecule. Specifically, a fixed hydrogen–oxygen–hydrogen angle value is imposed to ensure that the resulting linear quadrupole moment is equal to zero. This constraint is applied because the quadrupole moment is known to have minimal significance in the context of the model’s overall performance and accuracy.^28^ While both models have demonstrated similar accuracy in reproducing thermodynamic properties, they do exhibit some distinct characteristics. TIP3P-FB is characterized by a larger geometry, with a distance of 0.101 nm between the oxygen and hydrogen sites (*d*_OH_) and 0.164 nm between the hydrogen sites (*d*_HH_). Furthermore, the oxygen site in TIP3P-FB carries a partial charge of −0.848 e. In contrast, the geometry of OPC3 is smaller, with *d*_OH_ and *d*_HH_ values equal to 0.098 and 0.160 nm, respectively. Additionally, the oxygen site in OPC3 has a charge of −0.895 e. The variability observed between the optimized models may be attributed to an intrinsic limitation arising from the simplified description of the system. Furthermore, as both models are trained solely on average parameters derived from a top-down approach, it becomes intriguing to explore the potential advantages of integrating additional data on microscopic target features through a bottom-up approach.

In recent works, we introduced *Swarm-CG*,^7,10^ a versatile optimization software that is able to integrate bottom-up and top-down references in a multiobjective and multidirectional optimization framework for coarse-grained models. Building upon *Swarm-CG*’s capabilities, we propose a novel strategy for optimizing three-site water models by incorporating experimental data on the microscopic structure of water, particularly the radial distribution functions (RDF) of its atoms. Specifically, we utilize the oxygen–oxygen RDF (*g*_OO_), oxygen–hydrogen RDF (*g*_OH_), and hydrogen–hydrogen RDF (*g*_HH_) as the primary references for deriving our model. While our main objective is not to develop the most accurate three-site rigid model, we aim to explore the capabilities of *Swarm-CG* and assess the room for improvement in what can be considered *de facto* a coarse-grained description of water. The results we obtained are significant for two main reasons. First, we demonstrate that by selecting the optimization targets spanning different scales (micro + macro), such as the RDFs, density, and dielectric constant, it is possible to obtain an optimized water model with comparable accuracy to that of state-of-the-art models like TIP3P-FB and OPC3 while maintaining computational efficiency and robustness. Second, our findings allow us to investigate the chemical and physical origins that control the accuracy limits (indeterminacy) of model optimization. We investigate how these limits are intrinsic and are connected to the physical constraints of the model itself. The insights gained from this study hold significance not only for optimizing the specific system presented in this paper but also for any approximated model that relies on higher-accuracy data or incorporates top-down constraints based on experimental evidence.

## Methods

**Figure 1:**
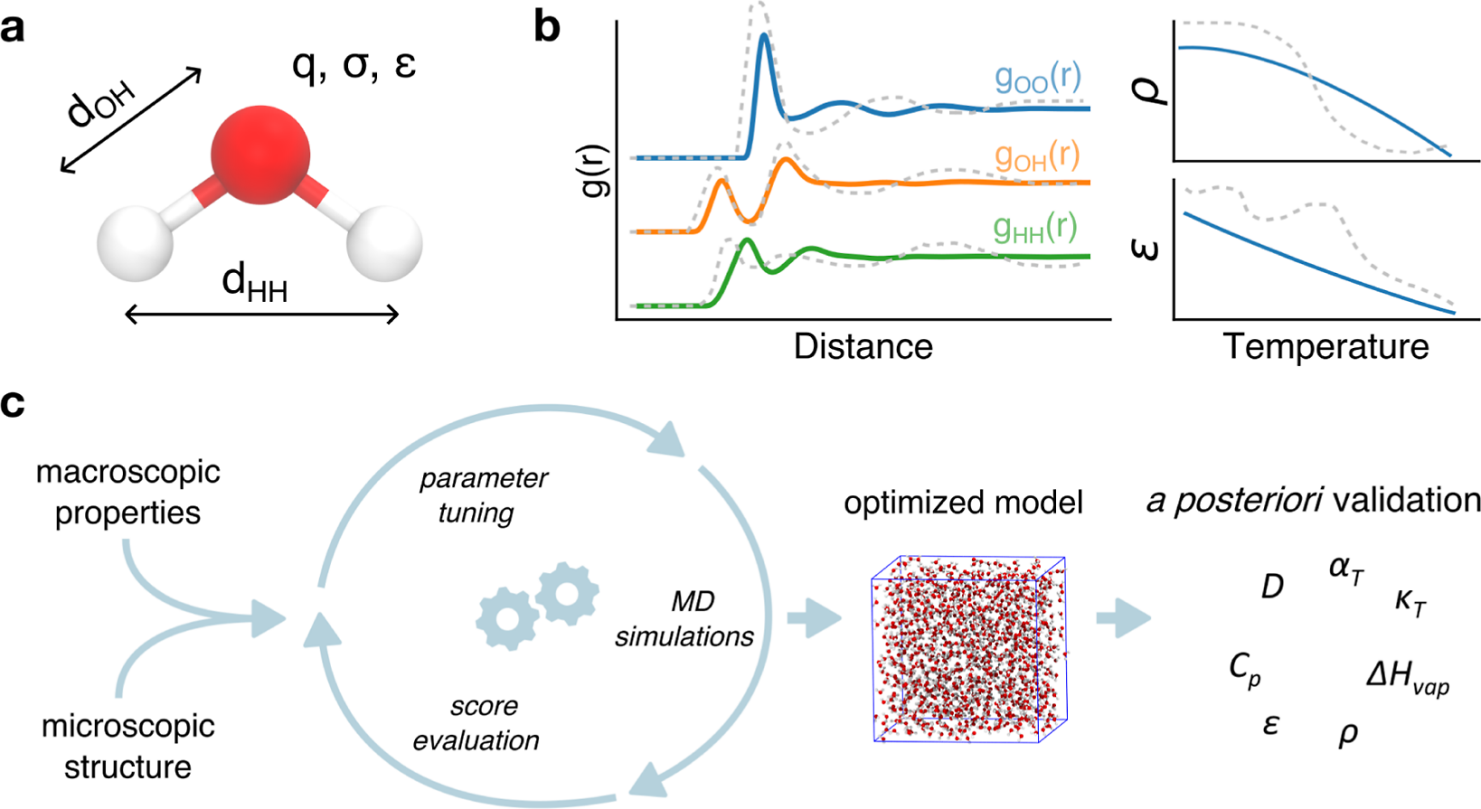
Overview of the study. (a) Representation of a water molecule and schematic of the five parameters that define the three-site model. (b) Experimental data used in this work: radial distribution functions *g*(*r*) (bottom-up reference), liquid water density (ρ) as a function of temperature, and static dielectric constant (ε) as a function of temperature (top-down references). (c) Workflow diagram illustrating the process of the study. Reference experimental data serve as targets guiding the optimization process. Swarm-CG runs iterative MD simulations, adjusting the parameters of the water molecule to reach the best match with the reference experimental data. The resulting optimized model is then evaluated and validated *a posteriori* against a set of experimental observables at different temperatures not in the training set.

The optimization work conducted herein builds on a multireference particle swarm optimization software that we developed recently: *Swarm-CG*.^7,10^ In particular, *Swarm-CG* has been developed to optimize bonded and nonbonded parameters in molecular models to fit experimental results (top-down references) and the behavior seen in all-atom MD trajectories (bottom-up references). *Swarm-CG* has been successfully tested to optimize a variety of molecular systems (e.g., lipid models^27^). In this paper, *Swarm-CG* has been adapted for this specific case study (a dedicated variant can be found at: https://github.com/GMPavanLab/wateropti). The five parameters of a general three-site rigid water model that are iteratively tuned (illustrated in Figure 1a) are (i) the intramolecular distance between the oxygen and the hydrogen sites, *d*_OH_, (ii) the intramolecular distance between the two hydrogen sites, *d*_HH_, (iii) the absolute charge of the oxygen site, *q*, and the two functional parameters of the Lennard–Jones potential, which is centered on the oxygen site, namely (iv) sigma σ and (v) epsilon ε. To achieve this objective, *Swarm-CG* relies on a population-based global optimization algorithm inspired by the collective movement of birds flocks and fish schools, specifically Fuzzy Self-Tuning Particle Swarm Optimization (FST-PSO).^29^ In PSO algorithms, a swarm of individuals (referred to as a swarm of “particles”) moves iteratively inside a bounded search space and cooperates to identify the best solution for a problem according to a scoring function. In FST-PSO, fuzzy rules allow to self-tune the hyper-parameters of the PSO algorithm during optimization, which improves its performance.^29^ Each particle of the swarm holds a set of values to be optimized and represents a distinct putative force field for the water molecule. Classical MD simulations are conducted automatically at each iteration of the algorithm using the iteratively refined force field parameters, and our scoring function evaluates the deviation of the models from target properties. These particles iteratively refine their positions in the parameter space (namely, they fine-tune the water model), according to the global best solution discovered by the swarm of particles. This iterative process continues until a predefined termination criterion is met, such as the achievement of a satisfactory solution. Population-based algorithms such as FST-PSO are efficient for global optimization tasks such as those at hand in this study. Here, we initialize the swarm of particles randomly within predefined boundaries of the force field parameter space (five dimensions), which enables its thorough exploration and mitigates premature convergence problems to local optima. In our case, we have continuated the optimization until all particles converge at the same point in the parameter space (*e.g.,* they propose the same water force field), and no substantial changes are made within the iterations. *Swarm-CG* exhibits inherent robustness to variations in the initial conditions due to the random distribution of particles in the parameter space at initialization. This stochasticity facilitates exploration across diverse solution regions, mitigating premature convergence to local minima. The algorithm further adapts parameters iteratively, guided by the fitness of the best particles (representing optimal force fields) acting as attractors for other particles.

We conducted our optimizations initializing swarms composed of 15 particles in the first and third subsections of the results and 26 particles in the second subsection. In each optimization procedure, a series of classical MD simulations are performed, and their discrepancy from the target properties is evaluated according to a scoring function (described below). Finally, the obtained optimized models are simulated at various temperatures across the liquid regime, and observables of interest are computed.

### Scoring Function

To quantify the discrepancy between the RDFs obtained from the simulations of the models vs the experimental ones from liquid water (at various temperatures), we introduced a scoring function based on the Earth mover’s distance (EMD) or Wasserstein distance.^30^ The Wasserstein distance is a measure of the dissimilarity between two probability distributions, based on the concept of optimal transport.^31^ It represents the minimum cost of transforming one distribution into the other, where the cost is proportional to the distance between pairs of points. In our case, we used the Wasserstein distance to compare the simulated RDFs with the experimental RDFs, with the distance matrix representing the differences between the radial distances of the bins of the distributions. In our work, we modified the standard computation of the Wasserstein distance by using the square of the distance matrix instead of the distance itself. Such a modification allowed to better account (weights more) the difference between the *g*(*r*) at a larger distance, which is important for capturing the long-range behavior of the water–water interactions and to avoid overfitting on short-range interactions. Preliminary tests demonstrated that this provided the best setup to compare *g*(*r*) curves as a whole in the most robust way. This modification also allows for mitigating potential problems emerging from the fact that classical three-site water models usually have difficulty reproducing the first peak of the RDFs (due to the fact that quantum effects are not included in the description of the system).^32^

In particular, the scoring function used in the optimization presented the first subsection of the results, which is
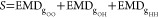
1where *S* represents the score and EMD_gOO_, EMD_gOH_, and EMD_gHH_ represent the Earth mover’s distance measurements of the three RDFs considered, namely, oxygen–oxygen, oxygen–hydrogen, and hydrogen–hydrogen, respectively. In this way, the scoring function does not capture discrepancies only in terms of distances and spatial displacement of the water molecules with respect to each other but also in terms of their natural orientation. This provides us with a scoring function that is rich in microscopic structural information on the system.

The optimizations presented in the second and third subsections of the Results and Discussion section involved not only fitting of microscopic features but also the density and static dielectric constant (macroscopic observables). The adopted score is expressed as
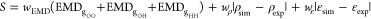
2where the first term represents the difference between the simulated and experimental RDFs for each type of particle–particle correlation. The second and third terms take into account the difference between the simulated and experimental values of density ρ and static dielectric constant ε, respectively. Each term in the score function has a weight assigned to it, which determines its relative importance in the optimization process. The weights were chosen as *w*_EMD_ = 0.5, *w*_ρ_ = 0.3, and *w*_ε_ = 0.2. Preliminary tests demonstrated that these weights ensured a balanced representation in the optimization process, allowing us to prioritize and place emphasis on fitting the RDFs over other macroscopic features of the systems. A comparison of experimental RDF with simulated *g*(*r*) examples scored according to our metrics is present in Figure S1 of the Supporting Information.

## Results and Discussion

This part is organized as follows. The first subsection presents the results of the model optimized to reproduce the experimental RDFs (*g*_OO_, *g*_OH_, and *g*_HH_) under standard conditions of 298 K and 1 bar. This approach focuses primarily on a pure bottom-up methodology, where the optimization is driven by the microscopic features of the water model. In the second subsection, we extend our analysis by optimizing the model to reproduce not only the RDFs but also the experimental density and static dielectric constant. Furthermore, the system is trained at two additional temperatures, specifically 280 and 343 K. This comprehensive optimization approach aims to capture a broader range of experimental observables, combining both bottom-up and top-down references. Finally, the last subsection provides a detailed investigation of the indeterminacy of the optimization problem within the context of the three-site representation.

### Multiobjective Optimization Based on Microscopic System Features

**Figure 2:**
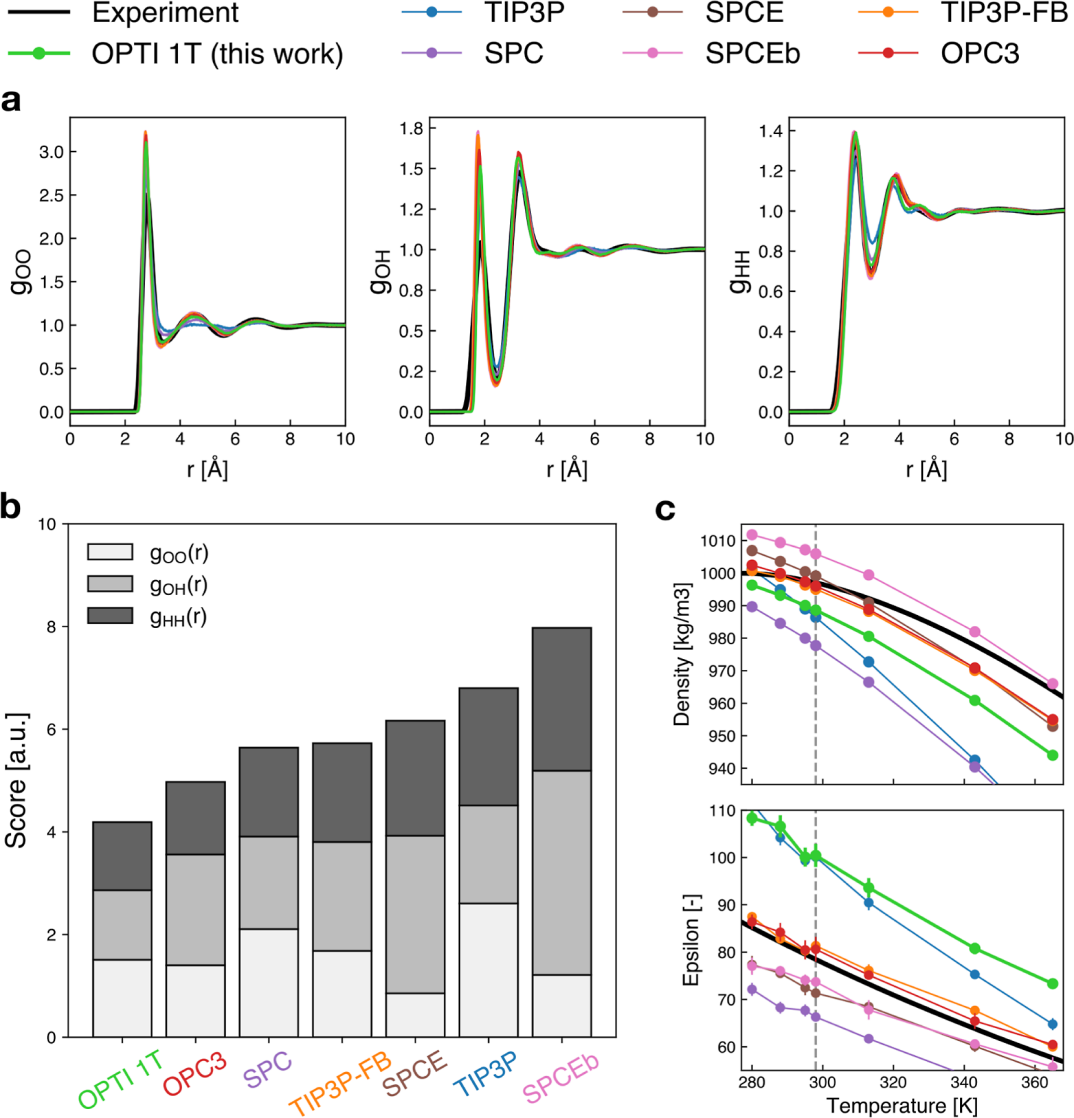
Results obtained from the first optimization, where the model OPTI 1T has been trained to reproduce experimental RDFs at 298 K and 1 bar. (a) RDF reproduction and comparison with other three-site water models: complete data (without superposition of curves) are provided in the Supporting Information (Figure S3). (b) Ranking of RDFs’ reproduction accuracy was based on our score. (c) *A posteriori* validation of the model with respect to density and static dielectric constant. Dashed vertical gray lines indicate the temperature at which the model was trained.

In a first optimization test, we trained the optimized water model according to a purely bottom-up approach to reproduce the experimental RDFs (*g*_OO_, *g*_OH_, and *g*_HH_) of water under the standard conditions of 298 K and 1 bar. At every iteration, *Swarm-CG* tests new parameters in the attempt to minimize the discrepancy between the *g*_OO_, *g*_OH_, and *g*_HH_ obtained from the model and the experimental ones under standard conditions. The obtained results are listed in Figure 2. A comparison with other popular three-site water models of the same type (OPC3,^33^ TIP3P-FB,^25^ SPC,^19^ SPCE,^34^ SPCEb,^35^ and TIP3P^18^) is also provided. A summary of the parameters for these models can be found in Table S1 of the Supporting Information.

According to the score that we formulated to quantify the deviation of the simulated RDFs from the experimental reference (eq 1), our model exhibited the highest level of accuracy in replicating the experimental RDFs (Figure 2b). Despite the fact that a model optimized as such is found to be the best one, this is not surprising since our model was optimized to reproduce experimental RDFs. Nonetheless, it can be noticed that also in our case, the oxygen–hydrogen RDF is overlocalized, which appears as unavoidable in such models, where the nuclear quantum effects are not explicitly included. Similarly, the attempt to fit at best the second and third solvation shell in the oxygen–oxygen RDF (identified by the second and third *g*_OO_ peaks) produces an unavoidable overlocalization of the first peak (an enlarged plot of the RDFs around the solvation shells is provided for clarity in Figure S2 of the Supporting Information).

It is worth noting that even at the training temperature, the value of the dielectric constant predicted by our best model deviated from the target by a substantial amount. Such a lack of accuracy can be attributed to the fact that training the water model on RDFs alone does not provide sufficient information about the interactions between atoms. As a result, quantities such as the static dielectric constant, which depends on dipole fluctuations and is sensitive to the charges on the water model, are not reproduced accurately enough if the model is not trained to do so. This also means that although the RDFs are well reproduced, this is not a sufficient condition for macroscopic properties to emerge spontaneously in the system. These considerations motivated us to also incorporate additional top-down experimental targets into the scoring function. The results of this integration are illustrated in the following section.

In terms of computational time, the refinement of five parameters of the water model at the single temperature condition required 4 days (wall-clock time) to reach convergence (500 swarm iterations) using 26 particles in the swarm and using 36 CPU cores, each simulation running on nine CPU cores equipped with a GPU.

### Multiobjective Multitemperature Optimization Based on Microscopic and Macroscopic Observables

**Figure 3:**
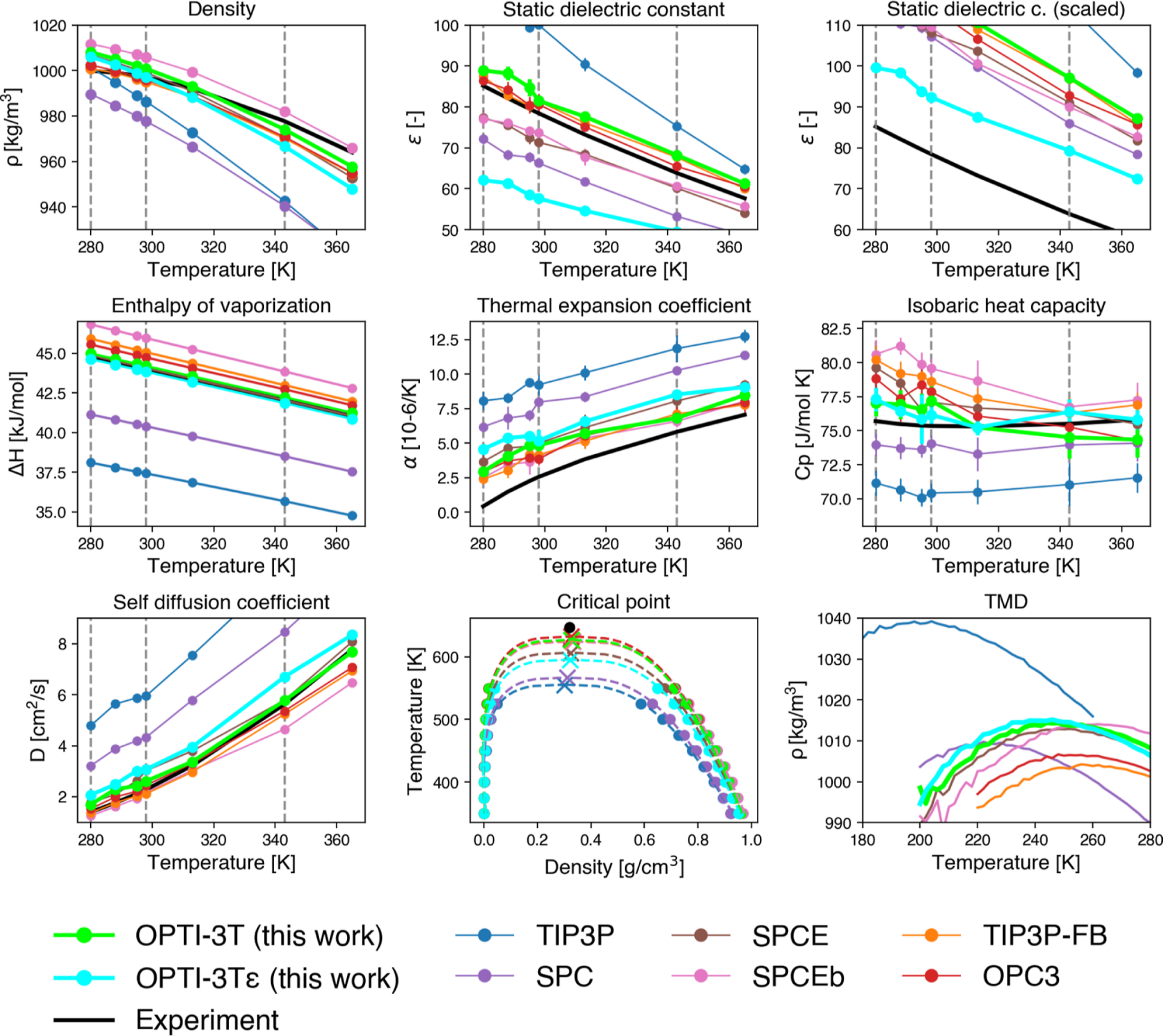
Performance of the OPTI-3T AND OPTI3Tε models and comparison with other three-site models against observables of interest. Reproduction of RDFs, isothermal compressibility, surface tension, and quantification of the deviation of simulated observables with respect to experimental data are provided in the Supporting Information (Figures S3–S6).

In a second test, we trained our water model using a hybrid approach, incorporating both top-down and bottom-up references, to attain an accurate reproduction of the experimental RDFs, density, and static dielectric constant at three distinct temperatures: 280, 298, and 343 K. This is thus a multitemperature multiobjective optimization combining top-down (microscopic) and bottom-up (macroscopic) target observables. For the optimized model (labeled as OPTI-3T), we assessed its performance in replicating key observables within the liquid phase. These included density, static dielectric constant, enthalpy of vaporization, thermal expansion coefficient, isobaric heat capacity, and self-diffusion coefficient (refer to Figure 3). Additional properties, such as isothermal compressibility, surface tension, and reproduction of the RDFs, are present in the Supporting Information (Figures S3–S5). The overall accuracy of OPTI-3T can be compared with the most advanced state-of-the-art data-driven trained models, such as TIP3P-FB^25^ and OPC3.^33^ In particular, worth noting is the agreement of our model with the experimental enthalpy of vaporization and self-diffusion coefficient at all of the explored temperatures. The enthalpy of vaporization reflects the strength of interactions between water molecules in the liquid state, representing the energy required to transition a molecule from the liquid phase to the vapor phase. On the other hand, the self-diffusion coefficient characterizes the dynamics of individual molecule diffusion within the liquid, indicating the ease of movement in a medium composed of other water molecules. Notably, our optimized model demonstrates remarkable agreement with experimental results for these two parameters despite not being explicitly targeted during training. This agreement underscores the significance of training the model on the RDF and these two additional macroscopic targets as they provide essential information for accurately reproducing fundamental thermodynamic and kinetic properties at the local level. A quantification of the accuracy of our OPTI-3T model by means of average deviation from the various experimental observables is present in Figure S6 of the Supporting Information. Overall, these results show the striking positive effect of training the water model based on microscopic information-rich observables (e.g., the RDFs) and how the microscopic characteristics of the model significantly influence most of its properties.

In the context of the development of empirical force fields, as underlined by Vega,^36^ a fundamental ill-posed assumption is typically made: namely, the charges used to calculate the potential energy surface (PES) are the same as those used to sample the dipole moment surface (DMS). This has led to the development of models matching experimental properties that depend directly on the PES (such as, e.g., density and enthalpy of vaporization) but that struggle to achieve an experimentally consistent reproduction of the static dielectric constant, both in the solid and liquid phase.^37^ In fact, the static dielectric constant depends on both DMS and PES, and the optimal charges used to describe the polarization of water may differ from those used to describe PES. A possible approach to tackle this problem, as shown in ref^38^, consists, e.g., in scaling the values of charges and dipole moment when computing properties that depend on the DMS (such as the static dielectric constant). The scaling factor λ is equal to
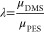
3where μ_DMS_ is the dipole moment of the DMS and μ_PES_ is the dipole moment computed with the charges used to sample the PES. The scaled static dielectric constant ε_λ_ is equal to
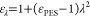
4where ε_PES_ is the static dielectric constant computed using the charges used to sample the PES. For sake of completeness, we thus performed another optimization under the same condition of OPTI-3T, this time accounting for corrections to the static dielectric constant following eq 4. In the context of our work, the term μ_DMS_ in eq 3 represents the experimental value of the water molecule immersed in the liquid phase and has been assumed equal to 2.9 D (following recent estimations^39,40^). The terms μ_PES_ and ε_PES_ are, respectively, the dipole moment and the static dielectric constant computed using the unscaled charges adopted during the simulation. In such a way, we are able to correct the values of the static dielectric constants of the models proposed by *Swarm-CG* during the optimization procedure. The model obtained in this optimization is labeled as OPTI-3Tε, and the results are showcased in Figure 3 alongside those of OPTI-3T.

The data obtained for OPTI-3Tε prove that a better match in reproducing the static dielectric constant is attained when scaled values are taken into account. Furthermore, the substantial offset of all three-site models observed in the scaled static dielectric constant illustrates the significant mismatch between optimal charges that fit the PES and the DMS. We have also calculated and tabulated (in Table 1) the dipole and quadrupole moments (calculated with PES charges) as well as the temperature of maximum density (TMD), melting temperature, and liquid–vapor critical point. For the models optimized in this work, the dipole μ is observed to be ∼2.3 D. This value assumes an intermediate position between the experimental value assumed by the molecule in the gas phase (∼1.8 D) and the one assumed in the liquid phase (∼2.9 D). The quadrupole moments *Q*_t_ and *Q*_0_ are known to significantly influence the water structure^41^ and phase diagram.^42^ For OPTI-3T, the quadrupole moments are similar to those of TIP3P-FB and OPC3. On the other hand, OPTI-3Tε presents a significant divergence in this respect, also exhibiting a negative *Q*_0_ value. This divergence can be attributed to the different methodology adopted during the optimization phase, where the decision to scale the dielectric constant caused a deviation toward quadrupole values that are outliers when compared to other models. Both the TMD and the critical temperature are verified to be lower than the experimental values for both optimized models. Such a discrepancy underlines an inherent limitation of three-site models in representing these properties. Although the dielectric constant constituted only 20% of the total optimization score, altering the optimization approach by scaling the dielectric constants exerted a substantial influence on the resultant models. This is particularly noticeable in the case of OPTI-3Tε, which adeptly reproduces the scaled dielectric, enthalpy of vaporization, and specific heat capacity (without considering dipole corrections, Figure S7). However, even in this scenario, these results demonstrate once more how improving the accuracy in reproducing certain properties has the counter-effect of a significant worsening in others (e.g., critical temperature and surface tension—see Table 1 and Figure S5 in the Supporting Information). Considering these observations, one can speculate that a parameter set for a three-site water model may only suboptimally fit both the PES and the DMS, and that there is a limit in the accuracy tightly related to the physical limits of these models.

**Table 1:** Comparison of Three-Site Models and Experimental Values for Linear Dipole Moment μ, Quadrupole Moments *Q*_t_ and *Q*_0_, TMD, Melting Temperature *T*_m_, Critical Temperature *T*_c_, and Critical Density ρ_c_^a^

model	μ [D]	*Q*_*t*_ [DÅ]	*Q*_0_ [B]	TMD [K]	*T*_m_ [K]	*T*_c_ [K]	ρc [g/cm^3^]
EXP	2.9^39^	NA	NA	277	273.15	647.1	0.322
SPC	2.27	1.97	0.00	222	190.5^43^	567	0.324
TIP3P	2.35	1.72	0.23	204	146(5)^44^	555	0.301
SPCE	2.35	2.04	0.00	248	214(3)^45^	607	0.324
SPCEb	2.37	2.08	0.00	262	224(4)	625	0.332
TIP3P-FB	2.42	2.05	0.07	256	216(4)^46^	626	0.329
OPC3	2.43	2.06	0.00	248	210(10)^47^	633	0.327
OPTI-3T (this work)	2.43	2.03	0.03	244	200(4)	627	0.327
OPTI-3Tε (this work)	2.28	2.10	–0.16	246	214(4)	596	0.322

a The values of uncertainty of TMD are ±1 K, and the values of uncertainty of critical temperatures and density are equal to ±4 K ± 0.02 g/cm^3^, respectively.

In terms of computational time, the refinement of five parameters of the water model at three levels of temperature required 8 days (wall-clock time) to reach convergence (300 swarm iterations) using 15 particles in the swarm and using 36 CPU cores, each simulation running on nine CPU cores equipped with a GPU.

These results give rise to several important considerations. First, the results obtained with our method demonstrate that, despite the fact that our models reproduce globally well the explored thermodynamic properties across the different conditions, the performances of OPTI-3T are not distant from those of, e.g., OPC3 and TIP3P-FB. This supports the hypothesis that, substantially, there is a limited room for radically improving the performances of three-site rigid water models. All our results suggest that there is an intrinsic limit in the accuracy that is achievable with models where the representation of the water molecule is so simplified. This leads us to fundamental questions. What are the key factors underpinning such limits? Are these imputable, e.g., to limitations in the optimization method itself or to intrinsic limits of the model? In the next section, we will deeper investigate these questions, obtaining interesting insights.

### Intrinsic Physical Limits and Indeterminate Optimizations of Rigid Three-Site Water Models

Recently, Izadi et al.^33^ suggested that three-site water models somehow possess inherent accuracy limitations due to their oversimplified nature, which hinders their ability to achieve a complete and experimentally consistent reproduction of observables across the liquid phase. Nevertheless, a relevant question that remains unanswered is the precise reason behind this intrinsic limitation.

**Figure 4:**
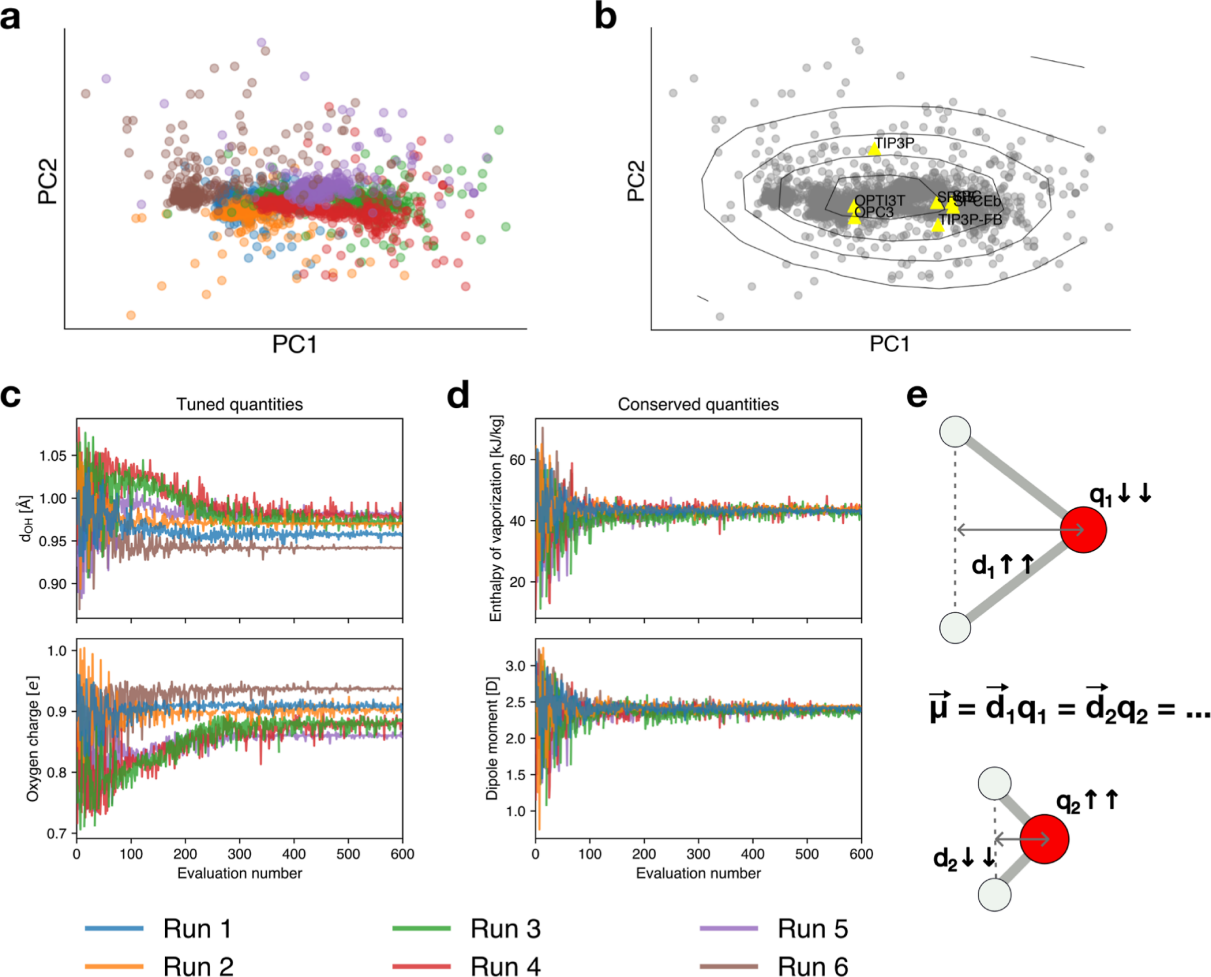
Results obtained by running a series of identical optimizations, initialized at different points of the parameters space. (a) Models obtained as solutions are displayed with PCA. Each color represents a different optimization run. (b) Density isolines. (c) Parameters that are tuned during the optimization—*d*_HH_ and oxygen charge—as a function of the number of iterations. (d) Example of quantities calculated *a posteriori* that are related to the energy of interaction between molecules—e.g., enthalpy of vaporization and dipole moment—as a function of the number of iterations. (e) Schematic representation of the solutions obtained from the series of optimizations: interdependence between size of the molecule and partial charges.

The performance of an automatic optimization procedure may be significantly influenced by a priori choices concerning the methodology and training variables. As a result, the ability of the model to accurately fit different observables may vary to some extent. In the case of OPC3, for example, it was considered crucial to impose constraints on the geometry of the molecules in order to ensure a quadrupole moment of zero.^33^ In the case of TIP3P-FB, a predominant emphasis was placed on a top-down approach, involving the simultaneous fitting to multiple thermodynamic observables across the liquid regime of water.^25^ To investigate the impact of these initial conditions and gain a comprehensive understanding of the optimization process, we conducted a series of six optimizations under identical constraints. Specifically, we minimized the discrepancy of RDFs, density, and static dielectric constant under the standard conditions of 298 K and 1 bar. Since the results obtained with such optimization cycles vary to some extent, this setup did not produce a single solution (identical in all six runs) but rather a group of solutions. Noteworthily, the obtained solutions demonstrate a comparable score (as illustrated in Figure S7 of the Supporting Information). Moreover, we conducted a principal component analysis (PCA) of all explored solutions achieved through the use of *Swarm-CG*. Figure 4a shows the high-density regions (i.e., the solutions projected on the first two principal components) that represent the optimal solution obtained from our optimization cycles. The different colors represent different runs. The contour lines illustrated in Figure 4b represent density isolines, which enable us to identify the regions of higher density points containing a set of optimal water models (according to our scoring function), which are characterized by a slightly different set of parameters. These data demonstrate how *Swarm-CG* brings the model systematically not to a specific solution (i.e., to a specific optimal model) but to a region of the space that contains “equally optimal, although slightly different solutions”. Interesting questions are, for example, why the method behaves in this way and specifically why slightly different solutions are “equally optimal”.

A deeper inspection of the “optimal solutions” provided by *Swarm-CG* revealed interesting patterns. In particular, it is interesting to observe that the dipole moments of all of the models belonging to this minimum is identical. Namely, despite the fact that their geometry or partial charges can be slightly larger/smaller (Figure 4c) in the various solutions, these change in such a way that the dipole moment the molecule is conserved. In such a way, the enthalpy of vaporization is also conserved across the various solutions (Figure 4d).

It is worth noting that these two properties (the dipole moment and the enthalpy of vaporization) are evaluated *a posteriori* and are not explicitly used to train the models during the optimization process. Moreover, both such observables are related to the extent of the intermolecular interactions, as the enthalpy of vaporization is proportional to the potential energy in the system and the interaction between dipoles of the molecules plays a key role in it. Figure 4d presents a visual representation of the variability in the charge and geometry of the water models generated by the different optimization runs. In qualitative terms, an increase in charge corresponds to a reduction in the size of the molecule, while a decrease in charge results in an increase in the size (Figure 4c,e). Additionally, we observed changes in the Lennard–Jones interactions with variations in sigma and epsilon values (Figure S8 in the Supporting Information). The obtained results and considerations illustrate how the collective properties of these models are largely controlled by the interplay of molecular dipoles and their interactions with each other.

Since in such a simplified three-site models, the majority of water–water intermolecular interactions are largely governed by the dipole moment, this introduces an intrinsic level of indeterminacy in the optimization. Recently, we have observed similar results also in the framework of the automatic optimization of, e.g., lipid models using *Swarm-CG*, where a certain level of model accuracy can be achieved, although accompanied by an inherent uncertainty. While uncertainties can arise from various sources in automatic approaches, such as the number of objectives, parameter selection, and optimization methods, it is worth noting that the uncertainty we are referring to in this context originates elsewhere. Here, due to the simplified physical description of the three-site rigid water model, the optimization problem becomes inherently undetermined as it seeks to find an optimized dipole, which is a composite variable represented by the product of charge and geometry (μ = *qd*). This leads to a degeneracy characterized by different optimal solutions with varying combinations of charges and geometry. To overcome this limitation, a potential improvement could involve incorporating additional parameters that decouple the geometric and electrostatic characteristics of the water model during the training process. For example, one approach could be training the model to reproduce a specific geometry obtained with higher accuracy from quantum mechanical (QM) approaches or calculating the electrostatic potential. However, implementing such an approach encounters challenges due to the substantial differences between the geometric and electrostatic descriptions at the QM level compared to the all-atom models, as demonstrated by recent research on OPC3.^26^

An interesting outcome of these considerations is that while our approach can achieve optimal solutions at least as good as the state-of-the-art models in a very efficient way, it also underlines how such all atom water models are *de facto* a coarse-grained description of the real water molecule features. Like other coarse-grained models, they encounter degeneracy due to simplified representations of system degrees of freedom, resulting in a certain level of precision combined with inherent indeterminacy. This indeterminacy implies that different parameterizations can lead to similar behaviors. Notably, in our case, this degeneracy yields a set of nonidentical solutions that belong to a minimum that is identified by the PCA.

These considerations suggest that similar principles used to parameterize other three-site rigid water models generally encounter similar limitations (i.e., the challenges and constraints faced in developing and optimizing water models based on similar principles are likely to be shared). To reduce the uncertainty in the model outcomes, another possible solution is to employ extra sites in the model, such as in the four-site models like TIP4P^18^ or TIP4P-ICE.^48^ Such an addition would permit us to expand the degrees of freedom to tune, allowing for a better fit of some properties, e.g., the curve of density across different temperatures. *De facto*, this underlines how, to improve substantially the performances of three-site water models, it is necessary to increase the resolution of the model, accounting for more degrees of freedom.

## Conclusions

In this work, we explored the effect of combining microscopic- and macroscopic-rich information into a training set of experimental observables used to automatically optimize classical three-site water models. In particular, as the microscopic target observables, we use the experimental *g*_OO_, *g*_OH_, and *g*_HH_ RDFs of liquid water at various temperatures that, altogether, contain information not only of how strongly the water molecules interact but also on how the molecules are organized in space with respect to each other. A first optimization of the water model under standard conditions (298 K and 1 bar) using only a bottom-up (microscopic) reference demonstrated how such microscopic information alone is insufficient to obtain an experimentally consistent reproduction of all other screened macroscopic observables for the liquid phase of water, especially for what pertains to the density and dielectric constant of liquid water.

Including in the training set and in the score, in a second test, the density and static dielectric constant of liquid water were then seen to provide considerable improvements. The obtained model showed a remarkable improvement in reproducing macroscopic properties, especially with respect to the self-diffusion coefficient and enthalpy of vaporization. This also suggests that these properties—density and dielectric constant—are not strongly dependent on the *g*(*r*) of water. Overall, we found that our optimized water model (called OPTI-3T herein) exhibits a comparable level of accuracy to that of the two models OPC3 and TIP3P-FB, which were also obtained through automatic optimization approaches and are considered state-of-the-art models in the realm of three-site rigid water models. Nonetheless, it is worth noting how, in our case, combining microscopic and macroscopic target properties allows for achieving such a level of accuracy in an efficient way and with a relatively reduced computational time (e.g., TIP3P-FB is trained on a large amount of thermodynamic properties at various temperatures in a computationally intensive process). At the same time, our tests show that there is little room for further improvement in these models by, e.g., adding more experimental observables in the training set, etc., which suggested that all these models are somewhat very similar and possibly nearly consistent with each other, considering the precision that it is reasonable to expect from them. Moreover, the calculation performed using the new estimate of static dielectric constant returned a new model that was improved in some observable and worse in others, corroborating the intrinsic limitation of three-sites representation.

The series of optimizations that we conducted herein under identical constraints (namely, fitting the experimental RDFs, density, and static dielectric constant at standard conditions) shows that these models are somewhat intrinsically limited in their accuracy. The same is true in some sense concerning the determinacy of their optimization cycles. The results shown in Figure 4 show how many of the screened thermodynamic observables are controlled in these simplified water models by the water dipole (μ), which is a composite variable that depends on both the charge (*q*) and geometry (*d*) of the water model (μ = *qd*). This leads to inherent indeterminacy in the solutions that are systematically obtained. This means that different combinations of charge and size can correspond to equally optimal solutions toward fitting of the targeted properties. A set of optimal solutions is thus typically obtained in such automatic optimizations instead of a single specific one. The PCA data of Figure 4a,b show how all such obtained “optimal” solutions belong, in our case, to the same global high-density minimum. In Figure 4c,d, it is demonstrated how all the slightly different solutions belonging to such minimum represent nearly identical molecular dipole and enthalpy of vaporization. While the broadness of such minima could be interpreted, e.g., as being imputable to some kind of statistical error or limit in the particle swarm optimization method used herein, these results suggest that this is most likely related to an intrinsic indeterminacy in how the problem is posed. In particular, the degrees of freedom in such “coarse-grained” atomistic description of the water models are so limited that the optimization process degenerates, providing equally optimal solutions that are nonetheless different from each other.

These results are interesting because they demonstrate that when dealing with the optimization of approximated models, there will be inevitably intrinsic limits due to degeneration of the optimal set of parameters that satisfy the conditions that are posed. In such a case, further improvements cannot be achieved without introducing additional degrees of freedom that can decouple in some way such composite variables into the fundamental ones, allowing to fine-tune the model. One way could be, e.g., to add some higher (quantum) level additional constraint that allows to decouple the dependence on the charge (*q*) from that of geometry (*d*) in the solution. However, the geometry and electrostatics of QM water molecules are so different from those of these AA models that tests in this sense proved inefficient. In the case of the classical water models studied herein, reaching higher precision thus requires expanding the model’s representation by adding additional “classical” degrees of freedom, for example, allowing for a more flexible and accurate description of the system. This is exactly the case of the higher precision that can be achieved by, e.g., four- or five-site models.^49,50^ Moreover, altering the degrees of freedom can have important effects, for example, on subtle dynamical mechanisms associated with water reorientational dynamics, as recently shown.^51^ However, these results are also interesting for the development of approximate molecular models in general. Recently, we have observed similar intrinsic limitations also in the optimization of, e.g., coarse-grained models of a variety of other molecular systems.^7,10,27^ This observation serves as a valuable lesson for developing models of all kinds and not just in the context of water simulations. Such inherent limitations and these challenges encountered in optimizing approximated models demonstrate the importance of considering the complexity of the system being studied and the type of information lost with approximated molecular models. The integration of multiple references and, in particular, combining bottom-up and top-down microscopic/macroscopic-level information in the training set can improve the efficiency and robustness in the models’ optimization. Nonetheless, the results discussed herein also offer an unambiguous example of how understanding the physical limits of approximated models can provide precious knowledge for guiding future research toward more robust and reliable modeling approaches.

## Computational Details

### MD Simulations

All of the simulations have been conducted using GROMACS version 2021.5^52^ with the following protocol. The starting systems’ configuration is a cubic box containing 1024 water molecules, arranged in initial random configurations, using PACKMOL.^53^ After a preliminary energy minimization via steepest descent algorithm (for 2 × 10^3^ steps), the system is then equilibrated for 5 ns in the *NpT* ensemble and simulated for 10 ns in the same ensemble. Both these equilibration and production phases are simulated with a 2 fs time step. We kept the temperature constant with a velocity rescale thermostat^54^ (with a time constant of 0.2 ps) and the pressure constant to 1 bar with a cell rescale barostat^55^ (with a coupling constant of 1 ps and compressibility of 4.5 × 10^–5^ bar). A cutoff distance of 1 nm was used for short-range electrostatic and van der Waals interactions, and the long-range interactions were computed with the particle-mesh Ewald summation method.^56^ Corrections to long-range pressure and potential energy were considered.^57^

### Observables

#### Density

The mass density of water ρ was calculated as follows
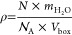
5where *N* is the number of water molecules (1024 in our case),  is the mass of water molecules in u.a.,  is Avogadro’s number, and *V*_box_ is the volume of the simulation box. Experimental reference data of ρ are taken from ref (58).

#### Static Dielectric Constant

We calculate the static dielectric constant from the fluctuations of the total dipole moment *M* of the simulation box, i.e., as
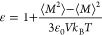
6where ε_0_ is the permittivity of the vacuum, *V* is the volume of the simulation box, *k*_B_ is the Boltzmann constant, *T* is the temperature of the system, and ⟨·⟩ represents the thermodynamic average. We calculated this observable using the routine gmx dipoles of the GROMACS suite. Experimental reference data of ε are taken from ref (58).

#### Radial Distribution Function

We calculated the RDFs of oxygen–oxygen, oxygen–hydrogen, and hydrogen–hydrogen [*g*_OO_(*r*), *g*_OH_(*r*), *g*_HH_(*r*)] pairs with MDAnalysis 2.0.0.^59^ We considered a cutoff of 10 Å and 500 equally spaced bins. Experimental reference data of RDFs are taken from ref (60).

#### Self-Diffusion Coefficient

The self-diffusion coefficient *D* is calculated using Einstein’s relation for a diffusive particle as
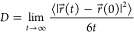
7where the quantity in the numerator is the mean square displacement (MSD), averaged over the trajectories of individual particles. Diffusion coefficients calculated with MD simulation are often referred to as *D*_PBC_, because they contain systematic errors due to the finite box size.^61^ Following ref (61), it is possible to correct this artifact obtaining the theoretical value of self-diffusion coefficient of water in an infinite box (*D*_0_). To this end, we calculated *D*_PBC_ in cubic simulation cells with *N* = 512, 1024, 2048, 4096, and 8192 water molecules. The protocol used for these simulations is identical to the one described at the beginning of this section, except for a different production time, i.e., 20 ns (*N* = 512), 15 ns (*N* = 1024 and 2048), and 10 ns (*N* = 4096 and 8192). We calculated the various *D*_PBC_ values using the gmx msd routine of GROMACS^52^ and *D*_0_ with linear interpolation. Experimental reference data are taken from ref (62).

#### Enthalpy of Vaporization

The enthalpy of vaporization Δ*H*_vap_ of 1 mol of liquid water in the gas phase can be approximated as^63^
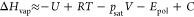
8where *U* and *V* are, respectively, the average potential energy and the volume of 1 mol of water molecules at pressure *p* and bath temperature *T*. *p*_sat_ is the value of the saturation pressure at temperature *T*. The term *E*_pol_ represents the depolarization energy of 1 mol of water molecules when it is transferred from the liquid to the gas phase.^34^ It can be expressed as
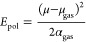
9where μ is the dipole moment of the simulated model, and μ_gas_ and α_gas_ are the dipole moment and average polarizability of a water molecule in the gas phase,^63^ respectively. The last term in eq 8 contains corrections that account for the vibrational effects of water molecules and nonideality of the gas phase. These corrections are reported in ref (63) for different temperatures. Experimental reference data are taken from ref (63).

#### Specific Heat Capacity

We computed the isobaric heat capacity *c*_p_ using the enthalpy fluctuation formula, namely
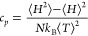
10

We computed this observable by using the gmx energy routine of the GROMACS^52^ suite. The value obtained was then corrected to account for quantum effects that are not considered in the classically computed heat capacity in eq 10. Specifically, these corrections include estimation of intramolecular vibrational energies (because our model is rigid) and intermolecular high-frequency modes. The values of these corrections are reported in ref (63) (Horn et al). The experimental reference data of *c*_p_ are taken from ref (64).

#### Thermal Expansion Coefficient

We calculated the thermal expansion coefficient α_T_ using the enthalpy–volume fluctuation formula
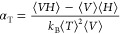
11

We computed this observable by using the gmx energy routine of the GROMACS^52^ suite. The experimental reference data of α_T_ are taken from ref (64).

#### Isothermal Compressibility

We calculated the thermal expansion coefficient κ_T_ using the volume fluctuation formula
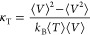
12

We computed this observable using the gmx energy routine of the GROMACS^52^ suite. The experimental reference data of κ_T_ were taken from ref (64).

#### Surface Tension

The interface between water and void was prepared and simulated following the good practices outlined in ref (65). First, a cubic box containing 1024 water molecules was equilibrated in the *NPT* ensemble. To represent the void phase, the *z*-axis of the simulation box was elongated by a factor of 4. The resulting biphasic system was then simulated for 50 ns in the *NVT* ensemble. The surface tension of the water–void interface was calculated using the mechanical or pressure approach,^66^ which involves evaluating the inhomogeneity of the pressure tensor as follows
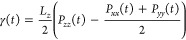
13where *L*_*z*_ is the elongation of the *z*-axis, and *P*_*xx*_, *P*_*yy*_, and *P*_*zz*_ are the diagonal components of the pressure tensor. To perform the analysis, we used the gmx energy routine of the GROMACS suite.^52^ Experimental reference data for the surface tension are obtained from ref (67).

#### Temperature of Maximum Density

To estimate the TMD, we conducted a series of *NpT* simulations with 1024 water molecules for 40 ns at different temperatures, incrementing every 2 K. We applied the same protocol described above for both the simulations and the estimation of density.

#### Melting Temperature

The melting temperature of ice *I*_h_ has been estimated from direct coexistence of the solid–liquid interface.^45^ The initial configuration of 1024 ice molecules have been obtained with the program GenIce,^68^ which generates a hydrogen-disordered lattice with zero net polarization satisfying the Bernal–Fowler rules. The solid lattice is equilibrated by performing a 10 ns anisotropic *NpT* simulation at ambient pressure (1 bar). On the other hand, the liquid phase is obtained from the same initial configuration of ice *I*_h_, performing a *NVT* simulation at *T* = 400 K in order to quickly melt the ice slab. The two phases are put in contact, resulting in a system of 2048 molecules in such a way that the solid face in contact with the liquid is the secondary prismatic 1210 plane. We then conducted a series of *NpT* simulations at different temperatures, controlling the pressure with Parrinello–Rahman barostat in its anisotropic version. The melting or freezing process is observed by monitoring the potential energy of the simulation system. In the direct coexistence simulation, when the temperature surpasses the actual melting point of the water model, the potential energy of the system rises until the entire box undergoes melting. Conversely, in simulations with temperatures below the melting points of the water model, the potential energy decreases until the entire box solidifies into ice. In the case of three-site models, this temperature is typically very low (200 K and less). The increase in potential energy indicating the melting of the system is observed on timescales of 10–1000 ns, depending on the temperature values. However, in the case of freezing, this process may take up to several microseconds to occur, likely due to the slow dynamics at such low temperatures.^45,47^

#### Critical Temperature and Pressure

The critical point for all models was determined using the direct coexistence method.^69^ Initially, we equilibrated a system of 8192 water molecules under different *NpT* conditions (1 bar and a temperature range from 350 to 550 K, incremented every 25K). To achieve this, we elongated the periodic box in one direction by flanking the original simulation box with two empty cubic boxes. Subsequently, constant *NVT* MD simulations were performed until an equilibrium was reached. We estimated the equilibrium densities of each phase by computing a density profile along the long side of the box. We utilized this collection of data of the liquid and vapor phases at various points to estimate the critical point using the law of rectilinear diameters and a three-term Wegner expansion of the form^70−72^
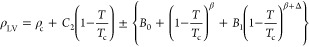
14where ρ_c_ is the critical density, *T*_c_ is the critical temperature, and *B*_0_, *B*_1_, and *C*_2_ are variable constants. The constants Δ and β were set to their standard values as established from renormalization group theory: Δ = 0.50 and β = 0.325.^73^

## Supporting Information Available

Additional details regarding the automatic optimizations and molecular simulations conducted (PDF)

## Notes

The input files, relevant analysis script, and the code use to conduct the optimization are available at https://github.com/GMPavanLab/wateropti. Complete data are also available at: 10.5281/zenodo.10257205. Other information needed is available from the corresponding author upon reasonable request.
