# Supplementary material for: Lessons Learned from Multiobjective Automatic Optimizations of Classical Three-Site Rigid Water Models Using Microscopic and Macroscopic Target Experimental Observables
Source: J Chem Eng Data. 2023 Dec 5;68(12):3228–41. doi: 10.1021/acs.jced.3c00538 (PMC10726314; doi:10.1021/acs.jced.3c00538)
Supplement: Supplementary file 1 — je3c00538_si_001.pdf [file je3c00538_si_001.pdf]

Lessons learned from multi-objective automatic optimizations of classical three-site rigid water models using microscopic and macroscopic target experimental observables

Mattia Perrone[1], Riccardo Capelli[2], Charly Empereur-mot[3], Ali Hassanali[4], and Giovanni M. Pavan[1,3,*]

[1]Department of Applied Science and Technology, Politecnico di Torino, Corso Duca degli Abruzzi 24, I-10129 Torino, Italy
[2]Department of Biosciences, Università degli Studi di Milano, Via Celoria 26, I-20133 Milano, Italy
[3]Department of Innovative Technologies, University of Applied Sciences and Arts of Southern Switzerland, Polo Universitario Lugano, Campus Est, Via la Santa 1, CH-6962 Lugano-Viganello, Switzerland
[4]The Abdus Salam International Center for Theoretical Physics, Strada Costiera 11, 34151 Trieste, Italy
[*]giovanni.pavan@polito.it

S1 Potential

The potential energy exerted on two interacting atoms i and j at separation r_{ij} is equal to:

$$U(r_{ij}) = U_C(r_{ij}) + U_{LJ}(r_{ij}) \ , \tag{S1}$$

where $U_C(r_{ij})$ is the Coulomb potential that accounts for electrostatic interactions and is equal to:

$$U_C(r_{ij}) = f \frac{q_i q_j}{\varepsilon_r r_{ij}} \ , \tag{S2}$$

where $f = \frac{1}{4\pi\epsilon_0} = 138.935458$, q_i and q_j are the charges of atoms i and j, ϵ_r is the dielectric of the vacuum (and is set to 1).
The term $U_{LJ}(r_{ij})$ is the Lennard-Jones potential is written as:

$$U_{LJ}(r_{ij}) = 4\varepsilon\left[\left(\frac{\sigma}{r_{ij}}\right)^{12} - \left(\frac{\sigma}{r_{ij}}\right)^{6}\right] \ , \tag{S3}$$

where ε is the depth of the potential well and σ represents the radius of the sphere that approximates the atom. The potential $U(r_{ij})$ does not contain a term that accounts for bonded interactions as they are kept fixed be means of the SETTLE algorithm.[1]

S2 Models compared in this work

Chemical name	CAS number	Model	Sigma [nm]	Epsilon [kJ/mol]	O charge (e)	O-H distance [nm]	H-H distance [nm]
Water	7732-18-5	SPC[2]	0.31655700	0.65062900	-0.820000	0.1000000	0.1633000
Water	7732-18-5	SPCE[3]	0.31655700	0.65062900	-0.847600	0.1000000	0.1633000
Water	7732-18-5	SPCEb[4]	0.31657195	0.64977520	-0.847600	0.1010000	0.1649300
Water	7732-18-5	TIP3P[5]	0.31506100	0.63638600	-0.834000	0.0957200	0.1513900
Water	7732-18-5	TIP3P-FB[6]	0.31779646	0.65214334	-0.848448	0.1011811	0.1638684
Water	7732-18-5	OPC3[7]	0.31742704	0.68369070	-0.895170	0.0978882	0.1598507
Water	7732-18-5	**OPTI 1T**	0.31921898	0.61796336	-0.935014	0.0940835	0.1518014
Water	7732-18-5	**OPTI-3T**	0.31657153	0.69750474	-0.889239	0.0977138	0.1590416
Water	7732-18-5	**OPTI-3Tε**	0.31584947	0.68161894	-0.874795	0.0980232	0.1631795

Table S1: Summary of the classical three-site rigid water models compared in this work and their forcefields parameters.

S3 Supplementary figures

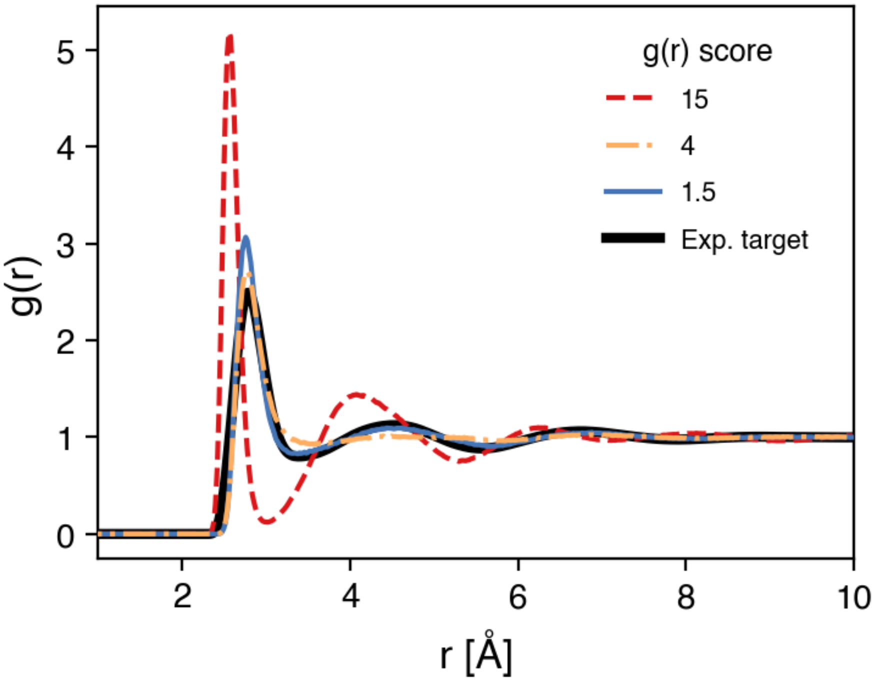

Figure S1: Comparison of experimental RDF and simulated RFDs examples scored according to our metrics. Notably, the orange curve receives a higher score (i.e., worse performance) compared to the blue one, despite capturing the first peak more accurately. The difference in scores arises from the orange curve's poorer reproduction of the second and third peaks. This observation highlights the scoring function preference for favoring long-range reproduction of RDFs in the evaluation of simulated curves.

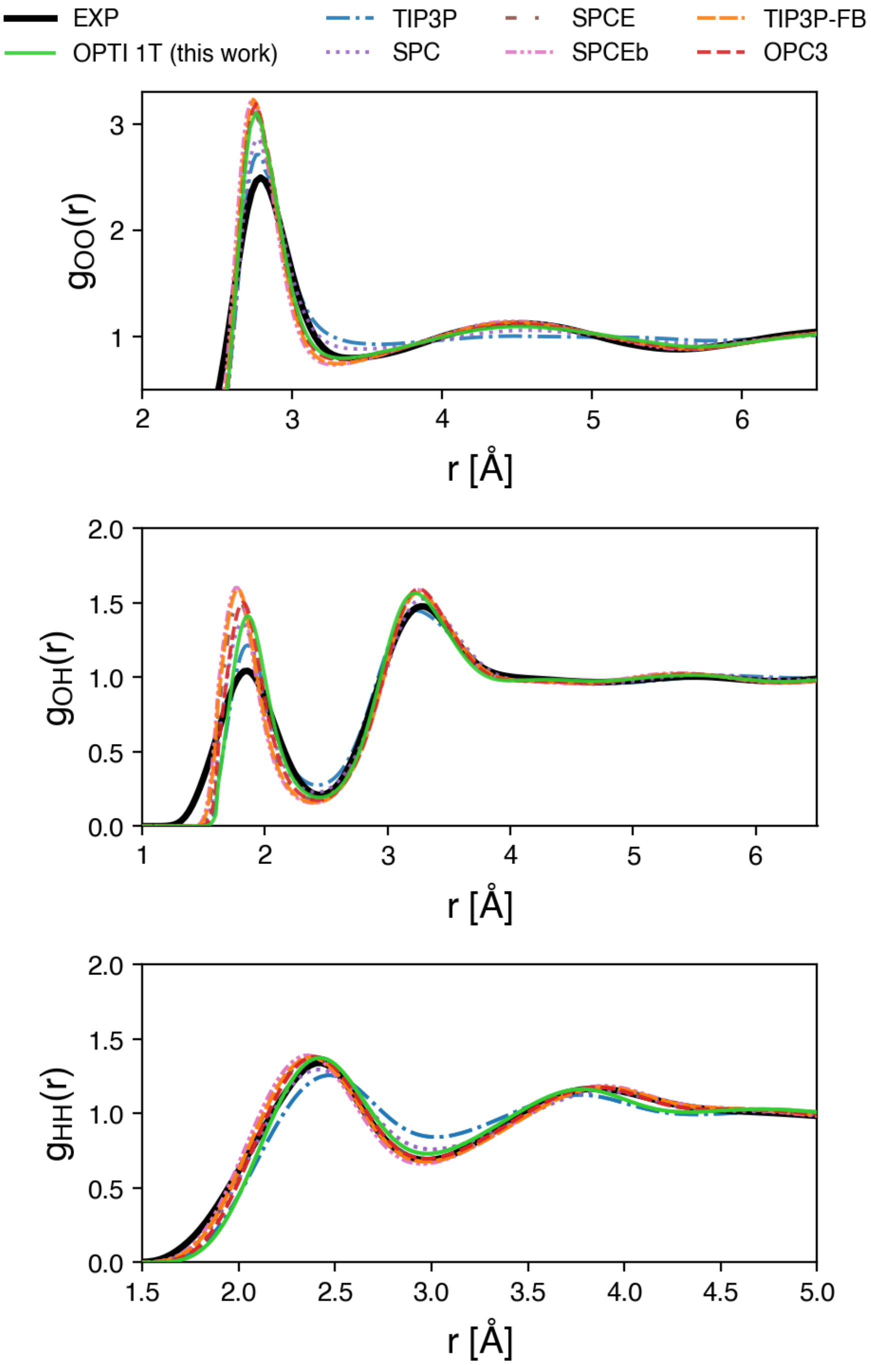

Figure S2: Enlarged plot of the reproduction of RDFs contained in Fig.2**a**

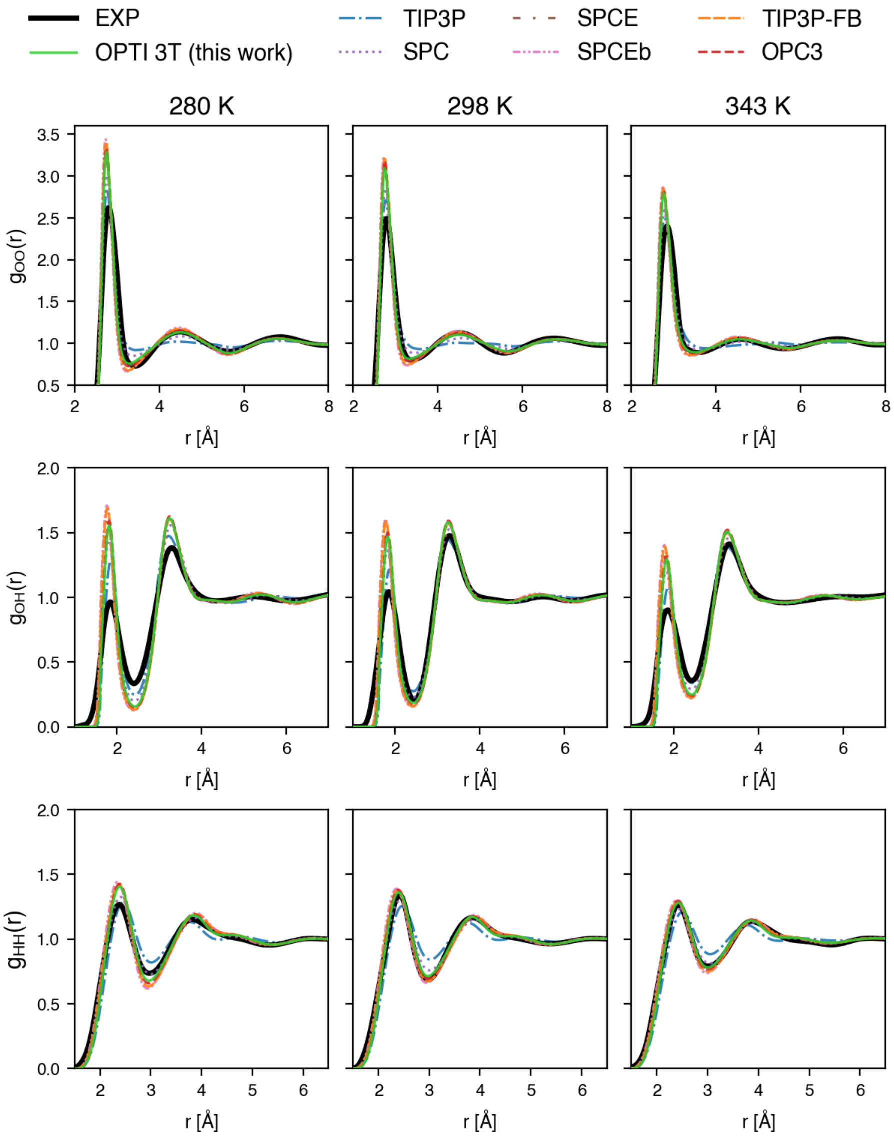

Figure S3: Radial distribution functions obtained of the model OPTI-3T along with a comparison with other models.

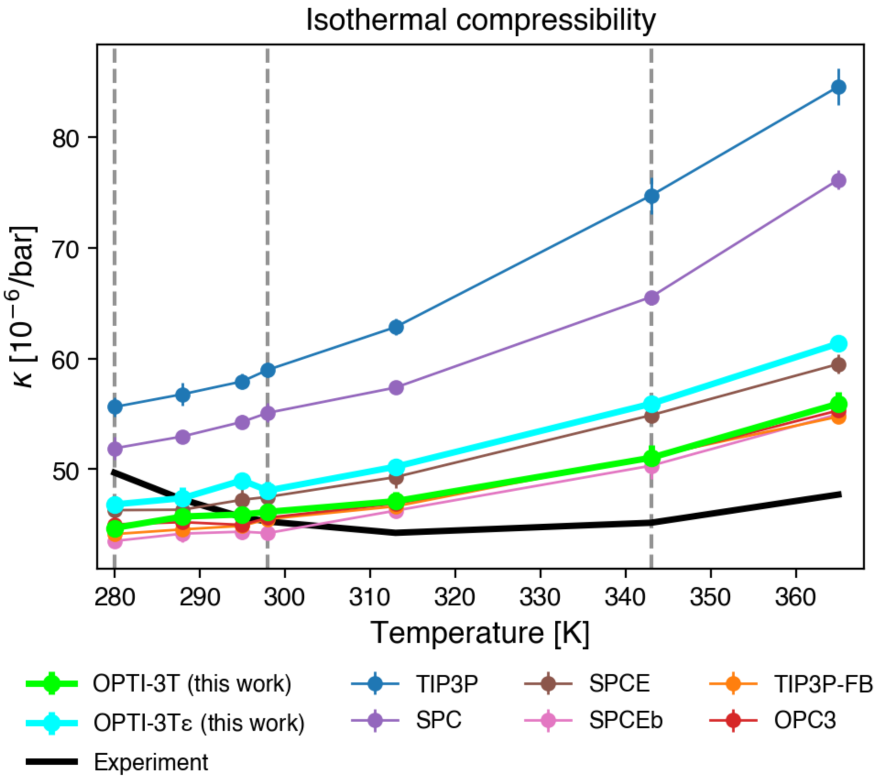

Figure S4: Isothermal compressibility as a function of temperature. Dashed vertical gray lines indicate the temperature at which the models were trained.

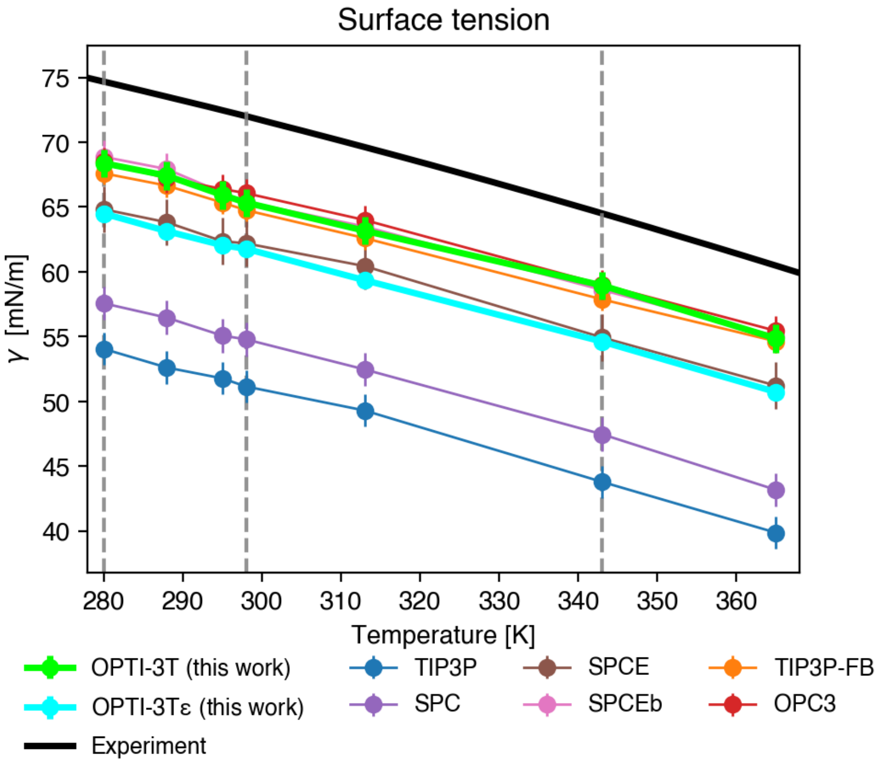

Figure S5: Surface tension as a function of temperature. Dashed vertical gray lines indicate the temperature at which the models were trained.

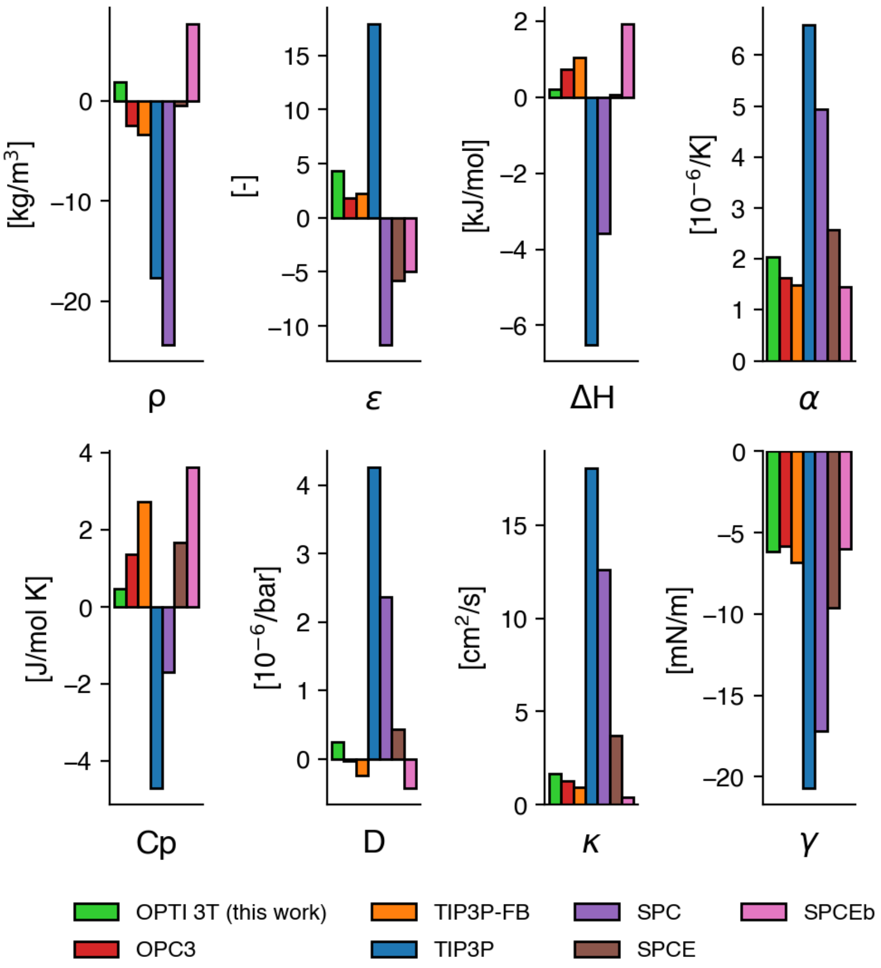

Figure S6: Average deviation of simulated observables with respect to experimental data in the liquid regime. The plots show water density, static dielectric constant, enthalpy of vaporization, thermal expansion coefficient, isobaric heat capacity, diffusion coefficient, adiabatic bulk modulus and surface tension.

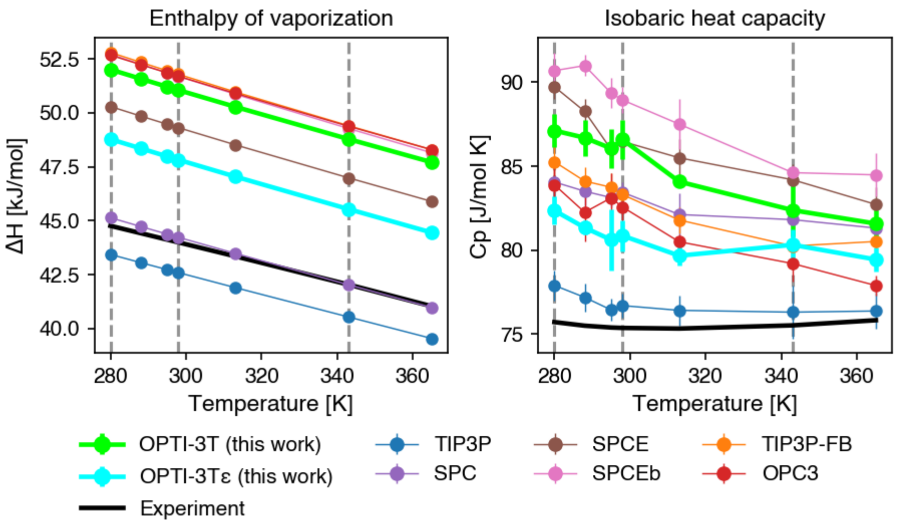

Figure S7: Plot of enthalpy of vaporization and isobaric heat capacity computed without accounting for polarization corrections. Dashed vertical gray lines indicate the temperature at which the models were trained.

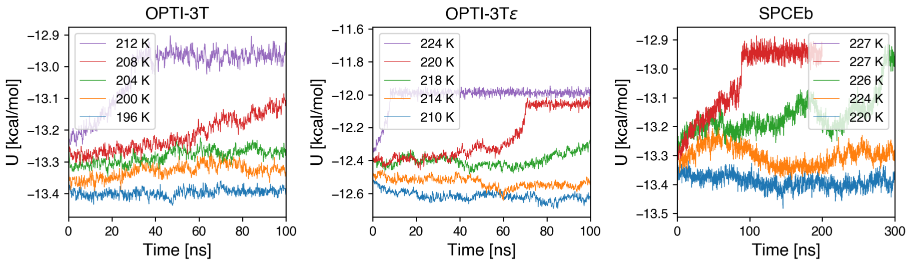

Figure S8: Evolution of the potential energy as a function of time of the liquid-ice coexistence simulation conducted at 1 bar and different temperatures. The models for which we estimated the melting temperature are OPTI-3T, OPTI-3Tε and SPCEb.

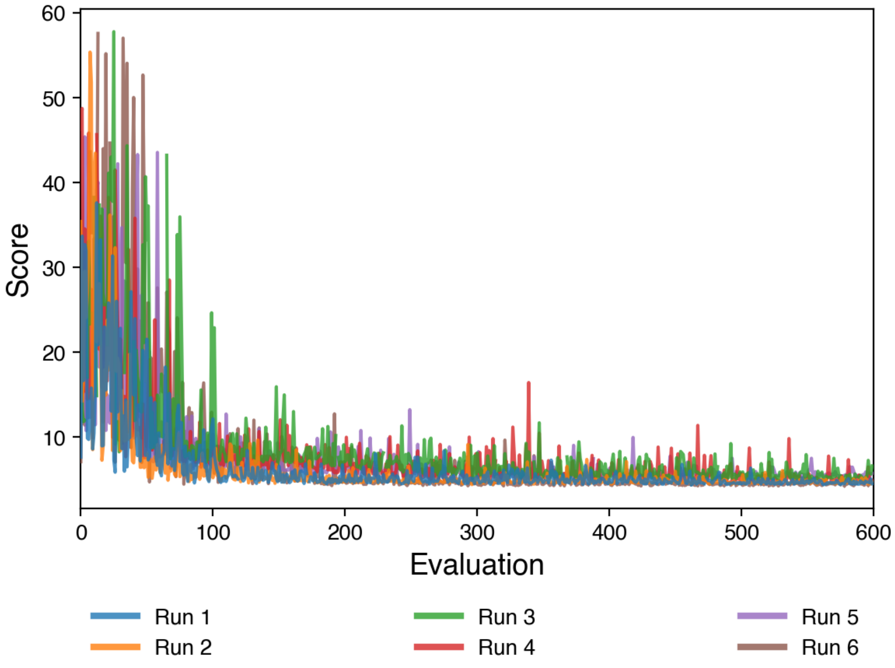

Figure S9: Score as a function of iteration number of the six optimizations. Each color represent an independent optimization run.

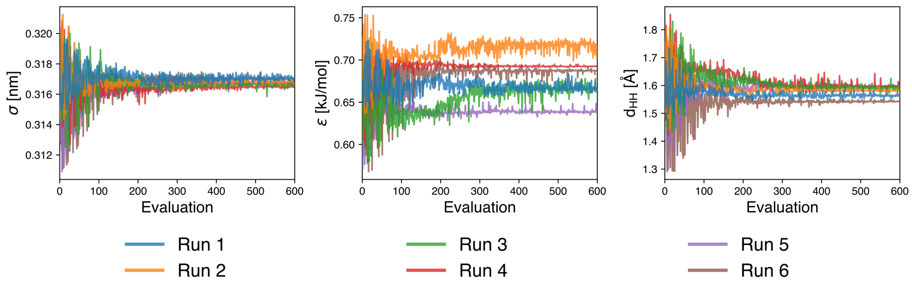

Figure S10: Values of sigma, epsilon and distance between the Hydrogens atoms as a function of iteration number. Each color represent an independent optimization run.

S4 Tabulated properties

The mean value of each point was determined from a simulation run of 100 ns. The standard error of the mean was computed using a block averaging approach, using five equispaced blocks of 10000 data points each, over the same 100 ns run. For the determination of the self-diffusion coefficient, the main paper outlines the procedure for estimating the extrapolated value by means of linear regression. The associated standard errors of the self-diffusion coefficient are derived by propagating uncertainties using the covariance matrix of the linear fit.

Model	280K	288K	295K	298K	313K	343K	365K
SPC	72.13(1.36)	68.27(1.26)	67.68(1.26)	66.28(0.62)	61.72(0.34)	53.23(0.74)	48.57(0.25)
TIP3P	111.50(1.46)	104.19(1.63)	99.34(1.06)	100.08(0.94)	90.46(1.61)	75.29(1.00)	64.76(1.27)
SPCE	77.36(1.85)	75.53(0.53)	72.48(1.61)	71.33(0.89)	68.48(0.59)	60.13(1.20)	54.10(0.66)
SPCEb	77.04(1.82)	76.05(1.06)	74.09(1.19)	73.72(0.39)	67.79(2.05)	60.61(0.53)	55.76(2.07)
TIP3P-FB	87.50(0.63)	82.83(0.15)	80.29(1.77)	81.36(1.07)	76.12(1.32)	67.67(0.79)	60.00(0.67)
OPC3	86.41(1.55)	84.21(1.97)	80.49(2.03)	80.62(2.63)	75.15(0.81)	65.43(1.66)	60.48(0.71)
OPTI-3T (this work)	88.92(0.75)	88.21(1.68)	84.76(2.04)	81.53(1.09)	77.65(1.31)	68.21(1.35)	61.26(1.37)
OPTI-3Tε (this work)	62.09(0.69)	61.36(1.19)	58.48(1.23)	57.62(1.07)	54.63(0.53)	49.52(0.70)	45.23(0.19)

Table S2: Computed values of water density expressed in kg/m^3.

Model	280K	288K	295K	298K	313K	343K	365K
SPC	73.96(0.79)	73.74(0.82)	73.63(0.58)	74.06(0.61)	73.28(1.24)	73.95(0.92)	74.08(1.34)
TIP3P	71.16(0.91)	70.66(0.83)	70.10(0.66)	70.43(0.70)	70.51(0.90)	71.06(1.57)	71.55(1.07)
SPCE	79.64(0.71)	78.50(0.72)	76.50(0.72)	77.09(0.70)	76.66(1.10)	76.33(0.92)	75.49(1.02)
SPCEb	80.60(1.09)	81.23(0.62)	79.86(0.90)	79.57(0.75)	78.67(1.50)	76.76(0.41)	77.25(1.29)
TIP3P-FB	80.22(1.04)	79.22(0.86)	78.99(0.82)	78.61(0.45)	77.37(0.77)	76.31(1.15)	76.89(0.90)
OPC3	78.84(1.16)	77.36(1.72)	78.36(1.46)	77.87(0.82)	76.07(0.43)	75.26(1.06)	74.27(0.61)
OPTI-3T (this work)	77.03(0.98)	76.93(1.08)	76.56(1.17)	77.21(1.19)	75.24(0.22)	74.52(1.55)	74.35(1.32)
OPTI-3Tε (this work)	77.30(0.84)	76.45(0.42)	75.86(1.80)	76.16(0.93)	75.24(0.56)	76.38(0.94)	75.82(0.71)

Table S3: Computer values of the static dielectric constant.

Model	280K	288K	295K	298K	313K	343K	365K
SPC	6.19(0.24)	6.82(0.82)	7.03(0.39)	7.98(0.31)	8.36(0.38)	10.28(0.25)	11.38(0.35)
TIP3P	8.07(0.66)	8.28(0.38)	9.39(0.31)	9.23(0.74)	10.10(0.55)	11.86(0.96)	12.75(0.47)
SPCE	3.66(0.71)	4.66(0.53)	4.78(0.40)	5.00(0.13)	6.13(0.95)	8.06(0.61)	9.25(0.37)
SPCEb	2.49(0.45)	3.45(0.58)	3.63(0.88)	4.07(0.72)	5.36(0.31)	6.59(0.40)	8.04(0.52)
TIP3P-FB	2.39(0.30)	3.03(0.55)	4.19(0.50)	4.15(0.72)	5.15(0.56)	7.13(0.33)	7.79(0.14)
OPC3	2.92(0.71)	3.72(0.53)	3.93(0.23)	3.86(0.38)	5.57(0.46)	6.93(0.69)	7.94(0.70)
OPTI-3T (this work)	2.94(0.46)	4.06(0.88)	4.85(0.36)	4.84(0.52)	5.71(0.37)	6.83(0.31)	8.49(0.30)
OPTI-3Tε (this work)	4.57(0.29)	5.38(0.30)	5.52(0.37)	5.18(0.78)	6.57(0.34)	8.54(0.20)	9.06(0.20)

Table S4: Computed values of the thermal expansion coefficient expressed in $10^{-6}/K$.

Model	280K	288K	295K	298K	313K	343K	365K
SPC	41.15	40.81	40.52	40.40	39.78	38.51	37.54
TIP3P	38.10	37.80	37.53	37.42	36.85	35.67	34.76
SPCE	44.89	44.52	44.21	44.07	43.40	42.05	41.04
SPCEb	46.83	46.44	46.11	45.97	45.26	43.86	42.81
TIP3P-FB	45.93	45.54	45.22	45.08	44.38	42.99	41.97
OPC3	45.56	45.19	44.87	44.74	44.07	42.73	41.73
OPTI-3T (this work)	45.00	44.64	44.33	44.20	43.55	42.24	41.25
OPTI-3Tε (this work)	44.64	44.28	43.98	43.84	43.19	41.86	40.86

Table S5: Computed values of enthalpy of vaporization expressed in in kJ/mol. Estimated error is in the order of 0.01 kJ/mol.

Model	280K	288K	295K	298K	313K	343K	365K
SPC	3.22(0.09)	3.88(0.07)	4.20(0.10)	4.33(0.11)	5.79(0.17)	8.46(0.11)	10.90(0.21)
TIP3P	4.79(0.09)	5.64(0.21)	5.88(0.18)	5.96(0.08)	7.54(0.29)	10.92(0.12)	13.30(0.20)
SPCE	1.78(0.09)	2.09(0.08)	2.64(0.10)	3.03(0.12)	3.80(0.09)	5.79(0.15)	8.07(0.18)
SPCEb	1.26(0.07)	1.62(0.09)	1.94(0.10)	2.17(0.09)	3.05(0.12)	4.65(0.18)	6.48(0.19)
TIP3P-FB	1.38(0.02)	1.77(0.05)	2.04(0.08)	2.12(0.09)	2.98(0.03)	5.24(0.15)	6.94(0.17)
OPC3	1.54(0.09)	2.00(0.8)	2.23(0.09)	2.46(0.07)	3.31(0.10)	5.37(0.13)	7.08(0.16)
OPTI-3T (this work)	1.70(0.06)	2.31(0.09)	2.43(0.10)	2.62(0.07)	3.38(0.11)	5.77(0.09)	7.68(0.14)
OPTI-3Tε (this work)	2.08(0.07)	2.49(0.08)	3.02(0.08)	3.08(0.06)	3.96(0.10)	6.70(0.12)	8.35(0.16)

Table S6: Computed values of the thermal expansion coefficient expressed in cm^2/s.

Model	280K	288K	295K	298K	313K	343K	365K
SPC	51.88(1.04)	52.95(0.33)	54.27(0.62)	55.08(0.87)	57.37(0.54)	65.54(0.54)	76.14(0.89)
TIP3P	55.62(0.90)	56.77(1.05)	57.92(0.69)	58.96(0.67)	62.83(0.74)	74.71(1.68)	84.55(1.65)
SPCE	46.30(0.62)	46.33(0.37)	47.23(1.02)	47.49(0.76)	49.28(0.95)	54.86(0.60)	59.49(0.88)
SPCEb	43.51(0.33)	44.17(0.79)	44.35(0.69)	44.22(0.35)	46.25(0.39)	50.31(1.16)	54.96(0.18)
TIP3P-FB	44.13(0.87)	44.55(0.61)	44.88(0.92)	45.49(0.29)	46.64(0.58)	51.14(0.80)	54.74(0.37)
OPC3	45.01(0.65)	45.21(0.64)	44.96(0.24)	45.61(0.91)	46.89(0.55)	50.99(0.94)	55.30(0.48)
OPTI-3T (this work)	44.68(0.38)	45.72(0.55)	45.90(0.71)	46.14(0.71)	47.10(0.89)	51.02(1.16)	55.91(1.04)
OPTI-3Tε (this work)	46.81(0.76)	47.38(0.98)	48.99(0.32)	48.07(0.80)	50.21(0.43)	55.90(0.10)	61.34(0.45)

Table S7: Computed values of the isothermal compressibility expressed in $10^{-6}/bar$.

Model	280K	288K	295K	298K	313K	343K	365K
SPC	57.57(0.64)	56.46(0.81)	55.08(0.76)	54.79(0.34)	52.47(0.76)	47.47(0.71)	43.17(0.65)
SPCE	64.83(0.59)	63.82(1.10)	62.37(1.00)	62.20(0.40)	60.44(0.34)	54.94(0.57)	51.22(0.90)
SPCEb	68.88(0.85)	67.93(0.40)	66.11(0.57)	65.40(0.74)	63.49(0.21)	58.60(1.18)	55.04(0.62)
TIP3P	54.06(0.36)	52.61(0.53)	51.78(0.60)	51.15(0.41)	49.30(0.53)	43.79(0.26)	39.87(0.62)
TIP3P-FB	67.60(0.69)	66.65(0.69)	65.35(0.47)	64.76(0.57)	62.60(0.47)	57.88(0.91)	54.63(0.44)
OPC3	68.48(1.02)	67.22(0.36)	66.39(1.50)	66.07(1.01)	64.00(0.68)	59.02(1.06)	55.44(0.56)
OPTI-3T (this work)	68.38(0.74)	67.42(0.57)	65.93(0.47)	65.34(0.70)	63.17(0.39)	58.95(0.19)	54.86(0.54)
OPTI-3Tε (this work)	64.50(0.50)	63.14(1.19)	62.05(0.42)	61.78(0.64)	59.35(0.80)	54.62(0.26)	50.72(0.29)

Table S8: Computed values of the water surface tension expressed in mN/m.

Model	280K	288K	295K	298K	313K	343K	365K
SPC	989.662(0.080)	984.546(0.081)	979.989(0.082)	977.709(0.084)	966.499(0.086)	940.400(0.093)	918.368(0.098)
TIP3P	1001.211(0.083)	994.915(0.085)	988.976(0.087)	986.403(0.087)	972.694(0.090)	942.473(0.098)	917.568(0.103)
SPCE	1006.921(0.077)	1003.607(0.077)	1000.434(0.080)	999.155(0.079)	990.880(0.082)	970.705(0.088)	952.902(0.092)
SPCEb	1011.773(0.075)	1009.379(0.077)	1007.184(0.078)	1005.941(0.078)	999.448(0.082)	981.954(0.086)	966.022(0.090)
TIP3P-FB	1000.784(0.076)	998.982(0.075)	996.334(0.077)	995.041(0.077)	988.245(0.081)	970.117(0.085)	954.730(0.089)
OPC3	1002.472(0.075)	999.901(0.076)	997.335(0.076)	996.001(0.077)	988.842(0.079)	970.837(0.084)	954.995(0.089)
OPTI-3T (this work)	1008.141(0.076)	1005.125(0.077)	1002.183(0.078)	1000.823(0.078)	993.148(0.081)	974.089(0.085)	957.688(0.090)
OPTI-3Tε (this work)	1006.136(0.077)	1002.489(0.079)	998.878(0.081)	997.192(0.081)	988.293(0.082)	966.645(0.089)	947.918(0.093)

Table S9: Values of water density expressed in kg/m^3 for the models.

References

[1] Miyamoto, S.; Kollman, P. A. *J. Comput. Chem.* **1992**, *13*, 952–962.

[2] Berendsen, H. J.; Postma, J. P.; van Gunsteren, W. F.; Hermans, J. Interaction models for water in relation to protein hydration. Intermolecular forces. 1981; pp 331–342.

[3] Berendsen, H. J. C.; Grigera, J. R.; Straatsma, T. P. *J. Phys. Chem.* **1987**, *91*, 6269–6271.

[4] Takemura, K.; Kitao, A. *J. Phys. Chem. B* **2012**, *116*, 6279–6287.

[5] Jorgensen, W. L.; Chandrasekhar, J.; Madura, J. D.; Impey, R. W.; Klein, M. L. *J. Chem. Phys.* **1983**, *79*, 926–935.

[6] Wang, L.-P.; Martinez, T. J.; Pande, V. S. *J. Phys. Chem. Lett.* **2014**, *5*, 1885–1891.

[7] Izadi, S.; Onufriev, A. V. *J. Chem. Phys.* **2016**, *145*, 074501.
